# First genotyping of *Blastocystis* in yaks from Qinghai Province, northwestern China

**DOI:** 10.1186/s13071-019-3436-5

**Published:** 2019-04-16

**Authors:** Mei Ren, Jun-ke Song, Fan Yang, Min Zou, Pin-xue Wang, Dan Wang, Hui-jun Zhang, Guang-hui Zhao, Qing Lin

**Affiliations:** 1 College of Veterinary Medicine, Northwest A&F University, Yangling, Shaanxi 712100 People’s Republic of China; 2 State Key Laboratory of Plateau Ecology and Agriculture, Qinghai University, Xining, Qinghai 810016 People’s Republic of China

**Keywords:** Yak, *Blastocystis*, Prevalence, Subtype, China

## Abstract

**Background:**

*Blastocystis* is a common protist that can infect domestic and wild animals worldwide. Yak (*Bos grunniens*), an ancient species which can survive in alpine regions, has supplied necessities to local residents in plateau areas for generations. However, the infections with *Blastocystis* in yaks has been ignored for a long time. In the present study, the infections and genotypes of *Blastocystis* spp. in domestic yaks from Qinghai Province (northwestern China) were explored.

**Results:**

Of 1027 faecal samples collected from yaks in seven regions of Qinghai Province, northwestern China, the total prevalence of *Blastocystis* was 27.07% (278/1027) targeting the small subunit ribosome rRNA (*SSU* rRNA) gene. This protist was detected in yaks within each examined age group, geographical origin and season. Significant difference in prevalence was found in yaks from different geographical origins. The highest prevalence (48.94%) was observed in animals from Haixi county. Sequence analysis revealed three animal-specific subtypes (ST10, ST12 and ST14) of *Blastocystis* spp. in these yaks, with ST10 being the predominant subtype widely distributed in all investigated regions, seasons and age groups. Interestingly, this is the first report about subtype ST12 infecting yaks.

**Conclusions:**

To our knowledge, this is the first systematic report on *Blastocystis* prevalence in yaks from China, and the findings provide fundamental data for establishing effective control measures for this protist in yaks as well as other animals in China.

## Background

*Blastocystis* is a common anaerobic unicellular protist of animals and humans. Since its first observation in gastrointestinal tracts of humans in 1911 [1], human cases of *Blastocystis* infections have been reported in Asia, Europe, Africa, America and Oceania [2–12], with prevalences up to 100% in Senegal River Basin [13]. In addition, more than 50 animal species have been reported as reservoirs of *Blastocystis*, including non-human primates (NHP), birds, reptiles and ruminants, with a prevalence of 0.3–100% [14–26]. Although controversy on pathogenicity of *Blastocystis* existed, this parasite has been detected in patients with diarrhea and irritable bowel syndrome (IBS) [13, 27]. Moreover, the presence of *Blastocystis* has also been recently indicated as a possible indicator of intestinal health [4].

The yak (*Bos grunniens*), an ancient bovid species from the late Pliocene, is listed as one of the three major cold-resistant species (together with polar bears and Antarctic penguins) of the world, and more than 95% of its population (approximately 16.7 million) are from China [28]. Qinghai (northwestern China) is a Chinese province with the largest stock of yaks (approximately 5.9 million animals are reported), providing meat, dairy, dung, wool and other living necessities, and also serve as means of transportation for local residents [29]. To date, several pathogens (e.g. foot-and-mouth disease virus, bovine viral diarrhea virus, bluetongue virus, *Cryptosporidium* spp., *Enterocytozoon bieneusi* and *Toxoplasma gondii*) have been detected in yaks [30–35]. The production performance of yaks is seriously affected by these pathogens, leading to heavy economic losses [30–35]. Additionally, significant public health problems caused by zoonotic pathogens (e.g. *Cryptosporidium* spp., *E. bieneusi* and *T. gondii*) due to the close relationship between yaks and local people should not be neglected. In 2017, *Blastocystis* was found in six wild yaks from Xi’an Qinling Wildlife Park in Shaanxi Province, northwestern China, and two subtypes (ST10 and ST14) were identified [25]. In the present study, the prevalence and subtypes of *Blasstocystis* in yaks from Qinghai Province were determined on a large-scale by molecular methods based on the small subunit ribosomal RNA (*SSU* rRNA) gene.

## Methods

### Sampling

**Fig. 1:**
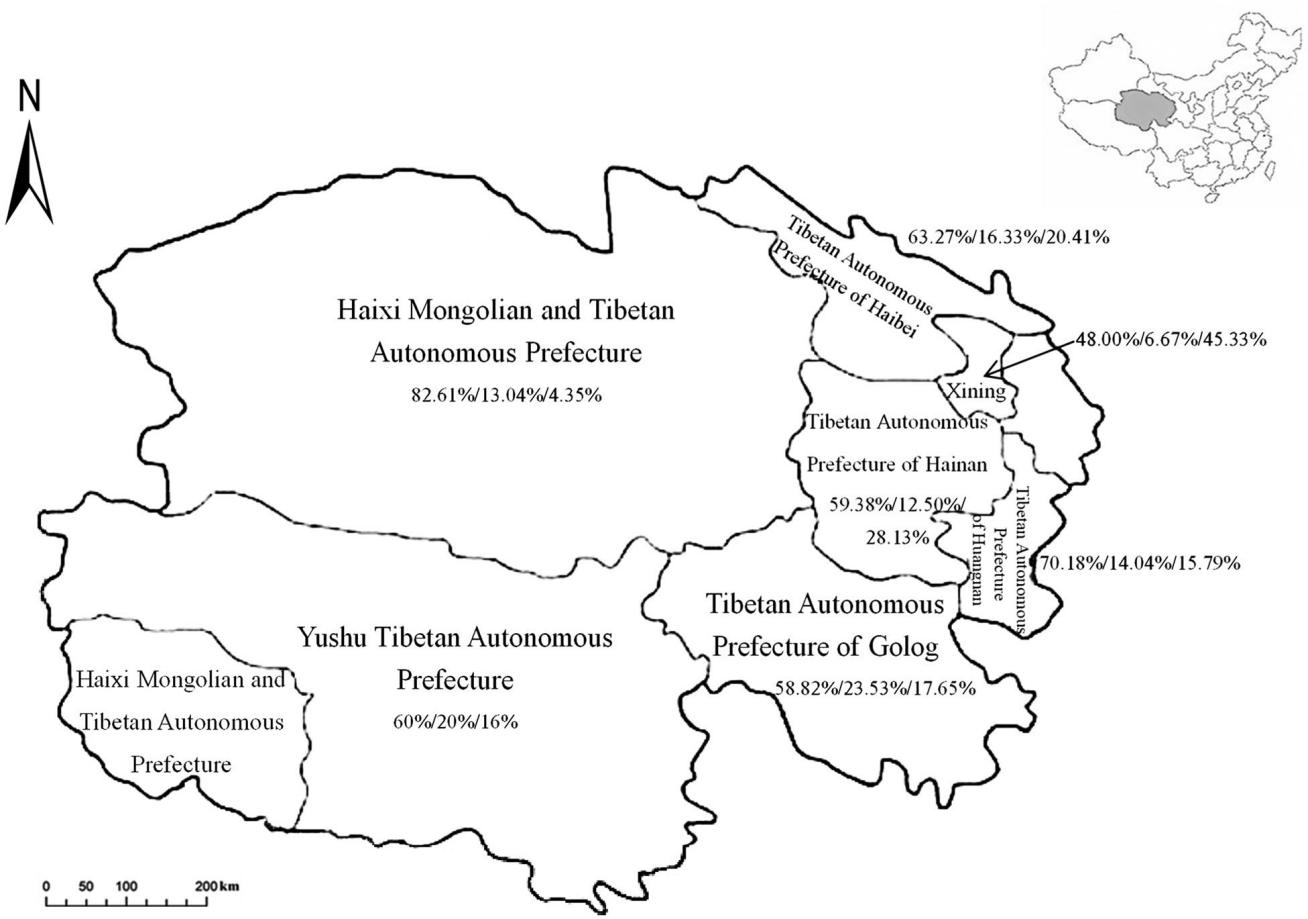
Geographical distribution of faecal samples from yaks in Qinghai Province, northwestern China. The proportion represents the frequency of each ST in the overall positive samples in certain location (ST10/ST12/ST14)

From May 2016 to October 2017, a total of 1027 fresh faecal samples were randomly collected from free-ranged yaks in 7 counties (Xining, Haibei, Golog, Hainan, Huangnan, Yushu and Haixi) of Qinghai Province (Fig. 1). Additionally, the altitude variation among these sites is 1980 m, with the highest spot in Golog (4473 m) and the lowest in Huangnan (2493 m). Each faecal sample was collected immediately after the animal excreted, placed in a plastic bag and marked with age, location and sampling date. Two age groups (> 6 months-old and ≤ 6 months-old) of yaks were determined by their dentition provided by yak owners, and each animal was only sampled once. All faecal samples were immediately transferred on ice to the laboratory, placed into 15 ml centrifuge tubes with 2.5% potassium dichromate, and stored at -20 °C for further study.

### Extraction of genomic DNA, nested PCR amplification and sequencing

Approximately 0.3 g of each faecal sample was washed with distilled water at 13,000× *g* for 1 min to remove potassium dichromate, and genomic DNA was extracted by commercial E.Z.N.A.^TM^. Stool DNA Kit (D4015-02) (Omega Bio-Tek Inc., Norcross, GA, USA) under the manufacturer’s instructions and then stored at -20 °C for further analysis.

Nested PCR was used to determine the presence of *Blastocystis* in faecal samples of yaks targeting the *SSU* rRNA gene with primers synthesized by ABI PRISM 3730 XL DNA Analyzer (Applied Biosystems, Carlsbad, USA), which were described previously [36, 37]. The reaction was performed in a 25 μl mixture containing 1 μl of genomic DNA, 2.5 μl 10 × Ex Taq Buffer (Mg^2+^ free), 2 μl dNTP Mixture, 1.5 μl MgCl_2_, 1 μl of each primer and 0.125 μl of TaKaRa Ex *Taq* (TaKaRa Bio Inc., Tokyo, Japan). The first amplification was implemented under the cycling condition previously described by Clark [36], while that of the secondary amplification was the same as the first one except that the first amplicon was used as template and the annealing temperature was 49 °C. All amplicons of the secondary amplification were examined by electrophoresis in 1% agarose gel with ethidium bromide.

All positive amplicons of the secondary amplification were sent to Sangon Biotech Co., Ltd., Shanghai (China) for sequencing.

### Sequence and phylogenetic analysis

The obtained sequence of each sample was assembled with the software DNAStar 5.0 [38], subsequently submitted to Basic Local Alignment Search Tool (BLAST) (https://blast.ncbi.nlm.nih.gov/Blast.cgi) for alignment with Clustul X 1.83 [39] and amended by the eye. To determine *Blastocystis* subtype, each of the corrected sequence was compared with the sequences from GenBank by BLAST analysis. The proofread sequences were then used to construct a phylogenetic analysis by the Neighbor-Joining (NJ) method within the software MEGA 7.0.26 [40]. Kimura 2-parameter model and bootstrap analysis (1000 replicates) were used [41], and *Blastocystis lapemi* (AY590115) was selected as the outgroup. In addition, Bayesian analysis (MrBayes 3.1.1) [42] was conducted with Hasegawa-Kishino-Yano 1985 (HKY) model of nucleotide substitution with four categories of among-site variation and the proportion of invariant sites, the best-fit model selected by jModelTest [43] using the Akaike information criterion (AIC) [44]. Bayesian analysis adopted four Markov chain Monte Carlo (MCMC) strands, 1,000,000 generations, with trees sampled every 100 generations. The tree was implemented in MEGA 7.0.26 after excluding an initial ‘burn-inʼ of 25% of the samples, as recommended.

### Statistical analysis

The differences in prevalence among different age groups, geographical origins and seasons were analyzed by Chi-square test/Chi-square goodness-of-fit test with the software SPSS 21.0 for Windows (SPSS Inc., Chicago, USA). The difference was considered statistically significant when *P* *<* 0.05.

## Results

### Prevalence of *Blastocystis* in yaks

In the present study, sequencing revealed that 278 faecal samples were positive for *Blastocystis* infection, with an overall prevalence of 27.07% in yaks from Qinghai Province (Table 1). *Blastocystis* was detected in the seven counties studied, and significant differences (*χ*^2^ = 35.652, *df* = 6, *P* = 0.002) in prevalence were found among these areas, with the highest prevalence in yaks from Haixi (48.94%) and the lowest in Hainan (19.75%) (Table 1). However, no significant differences of *Blastocystis* infection rates between seasons were detected (*χ*^2^ = 9.231, *df* = 3, *P* = 0.447) (Table 1). Furthermore, no significant differences between age groups were found (*χ*^2^ = 0.000005, *df* = 1, *P* = 0.556) (Table 1).

**Table 1:** Occurrence of *Blastocystis* from yaks in Qinghai Province

Factor	Category	Sample size	Prevalence (%) (No. of positive)	Genotype ST (*n*)		
				ST10	ST12	ST14
Age	≤ 6 months	48	27.08 (13)	9	0	4
	> 6 months	979	27.07 (265)	161	38	66
Season	Spring	215	22.33 (48)	26	7	15
	Summer	355	32.68 (116)	75	17	24
	Autumn	254	24.41 (62)	35	8	19
	Winter	203	25.62 (52)	34	6	12
Location	Xining	192	39.06 (75)	36	5	34
	Haibei	190	25.79 (49)	31	8	10
	Hainan	162	19.75 (32)	19	4	9
	Haixi	47	48.94 (23)	19	3	1
	Huangnan	270	21.11 (57)	40	8	9
	Yushu	90	27.78 (25)	15	6	4
	Golog	76	22.37 (17)	10	4	3
Total		1027	27.07 (278)	170	38	70

### Subtype analysis

**Fig. 2:**
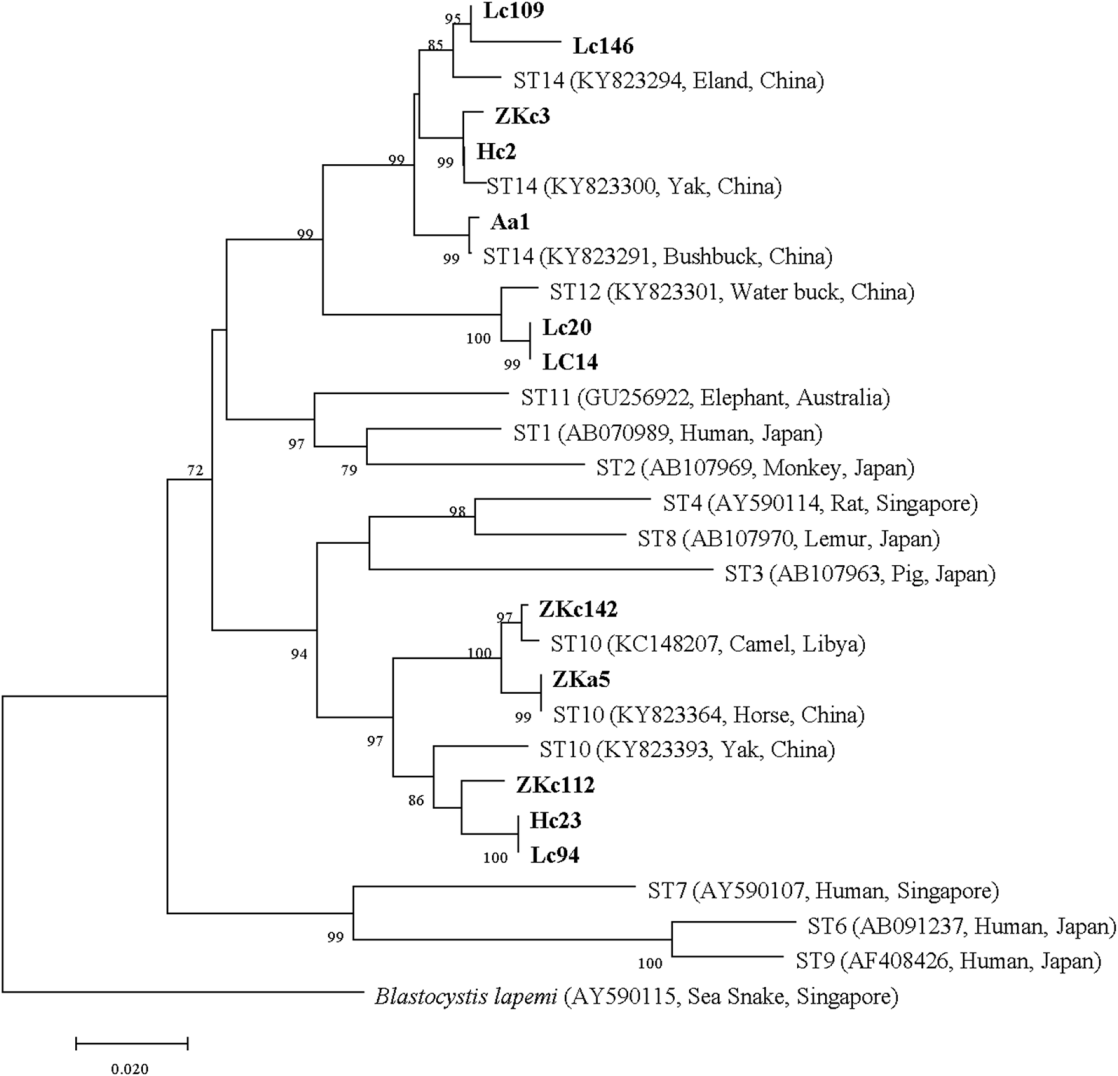
Phylogenic tree based on *SSU* rRNA gene sequences of *Blastocystis* using the Neighbor-Joining (NJ) method. New sequences are indicated in bold text. Only bootstrap values > 70% are shown (1000 pseudoreplicates). A sequence of *Blastocystis lapemi* (AY590115) was used as the outgroup. The scale-bar represents 0.02 substitutions per nucleotide site

**Fig. 3:**
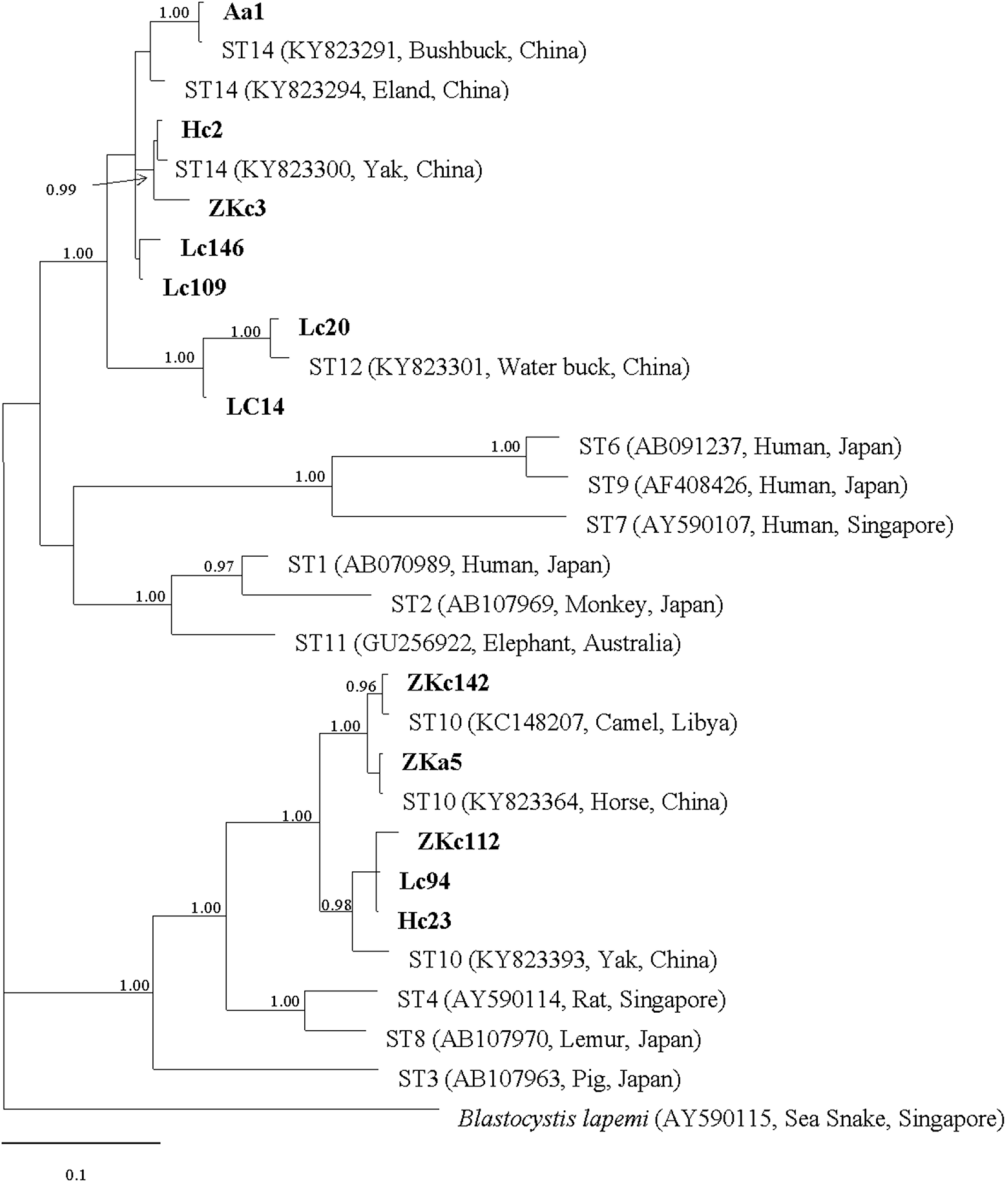
Bayesian analysis phylogenic tree based on *SSU* rRNA gene sequences of *Blastocystis* using Markov chain Monte Carlo (MCMC) method. Only posterior probabilities > 0.95 are shown. New sequences are indicated in bold text. The scale-bar represents 0.1 substitutions per nucleotide site

After amendment, sequences *c.*1000 bp long were used to reconstruct phylogenetic trees for subtyping. Of 278 *Blastocystis*-positive samples, three known *Blastocystis* subtypes were identified, including ST10, ST12 and ST14 (Figs. 2, 3). Among them, the subtype ST10 (61.15%, *n* = 170) was the predominant subtype, which was detected in all positive geographical regions, seasons and age groups, as well as the subtype ST14 (25.18%, *n* = 70). However, ST12 (13.67%, *n* = 38) was not found in yaks younger than six months (Table 1). No new subtype was found in this study as all sequences had homologies over 96% with published sequences on GenBank. In this sense, a previously proposed standard was used for naming a new subtype, as the nucleotide sequence divergences must be up to 5% [45].

## Discussion

*Blastocystis* spp. has been found worldwide in humans and several animal species [46]. In the present study, this enteric parasite was detected in domestic yaks from Qinghai Province. Previous studies have shown a prevalence of *Blastocystis* with a wide range of 0.3–100% in domestic and wild ruminants from several regions of Brazil, China, Colombia, Iran, Japan, Spain, Thailand and many other countries [17, 25, 47–51]. The overall prevalence (27.07%) in domestic yaks from Qinghai Province was within the range mentioned above; however, it was lower than that in captive yaks from Qinling Wildlife Park in Shaanxi Province [25]. The number of samples examined might result in these differences since in the study of Zhao et al. [25] only six captive yaks were examined, whereas in the present study 1027 faecal samples were examined.

Although in the present study no significant differences of *Blastocystis* prevalence were found between age groups, nor between sampling seasons, the infection status in the domestic yaks examined was significantly different depending on the geographical area of origin in Qinghai Province, with the highest prevalence (48.94%) in Haixi and the lowest (19.75%) in Hainan. Similar differences in the prevalence of this protist between regions have been observed previously in cattle from China [26]. Several reasons may lead to the prevalence differences, such as animal age, the different amounts of samples in different seasons, ecological environments, and one obvious difference among these seven sites which is the altitude variation mentioned above, caused a very different living conditions for both yaks and parasites [52].

Genetic variation has been reported in *Blastocystis* isolated from animals and humans worldwide [27, 46, 53, 54], and at least 17 known subtypes have been identified based on the sequence heterogeneity in the *SSU* rRNA gene [45, 54, 55]. Among these subtypes, ST10 was reported as predominant in species of the Artiodactyla [25]. Previously, in a study of wild animals in Qinling Mountains, two subtypes (ST10 and ST14) were found in captive yaks [25]. In the present study, these two subtypes were also detected in domestic yaks in Qinghai Province, suggesting a wide distribution of these subtypes. Additionally, subtype ST12 which was first detected in giraffes and kangaroos from Western Australia zoos in 2010 [56], was found in domestic yaks in Qinghai Province. As this subtype was mainly found in animals, like giraffes from Sydney Zoo (Australia. 2013) [57] and Qinling Wildlife Park (China, 2017) [25], waterbuck from Qinling Wildlife Park (China, 2017) [25], and cattle and goats from Thailand (2018) [50], it was not believed to be a zoonotic agent. However, subtype ST12 was identified in three stool samples in humans from Bolivia in 2016 [58]. The present study revealed for the first time the presence of subtype ST12 in the yak, indicating a possible transmission from yaks to humans which should be considered of great importance in the future as most herdsmen spend all the time with their yaks. Therefore, future study should be conducted to evaluate the zoonotic potential of this subtype.

## Conclusions

Collectively, the presence of *Blastocystis* was found in domestic yaks from Qinghai Province (northwestern China) with statistically significant variation of infection rates in relation to geographical origin. Moreover, susceptible populations should be alert because among the three subtypes found in this study, ST12 has been reported having a zoonotic potential in humans before, while ST10 and ST14 are considered infectious to animals only. To our knowledge, this is the first time ST12 infections are discovered in yaks.

## Abbreviations

*SSU* rRNA: small subunit ribosomal RNA
NHP: non-human primates
IBS: irritable bowel syndrome
BLAST: Basic Local Alignment Search Tool
NJ: Neighbor-Joining
HKY: Hasegawa-Kishino-Yano 1985
AIC: Akaike Information Criterion
MCMC: Markov chain Monte Carlo
